# Increased Preventive Effect on Colon Carcinogenesis by Use of Resistant Starch (RS3) as the Carrier for Polysaccharide of *Larimichthys Crocea* Swimming Bladder

**DOI:** 10.3390/ijms15010817

**Published:** 2014-01-09

**Authors:** Lian-Hong Chen, Jia-Le Song, Yu Qian, Xin Zhao, Hua-Yi Suo, Jian Li

**Affiliations:** 1 College of Life Science and Technology, Southwest University for Nationalities, Chengdu 610041, China; E-Mails: cdchenlianhong@gmail.com (L.-H.C.); lijian@swun.cn (J.L.); 2 Department of Food Science and Nutrition, Pusan National University, Busan 609-735, Korea; E-Mail: biopaul@pusan.ac.kr; 3 Department of Biological and Chemical Engineering, Chongqing University of Education, Chongqing 400067, China; E-Mail: foodmed@live.com (Y.Q.); 4 College of Food Science, Southwest University, Chongqing 400715, China; E-Mail: birget@swu.edu.cn

**Keywords:** polysaccharide, *Larimichthys crocea*, C57BL/6 mice, colon carcinogenesis, resistant starch

## Abstract

The preventive effect of polysaccharide of *Larimichthys crocea* swimming bladder (PLCSB) and the increase of this effect by use of resistant starch (RS3) as the carrier for PLCSB on azoxymethane (AOM) and dextran sulfate sodium (DSS)-inducing colon carcinogenesis in C57BL/6 mice has been studied. RS3 microspheres carrying PLCSB (RS3 + PLCSB) were produced and evaluated as a potentially improved colon carcinogenesis therapy for this study. The body weight, colon length, and colon weight of mice were determined, and colonic tissues were histologically observed. The serum levels of proinflammatory cytokines and the inflammation and apoptosis-related genes in colonic tissue were also tested. The PLCSB or RS3 + PLCSB significantly suppressed AOM and DSS-induced body weight loss, colon length shortening and decreased the colon weight to length ratio. PLCSB or RS3 + PLCSB reduced the levels of the serum pro-inflammatory cytokines IL-6, IL-12, TNF-α, and IFN-γ to a greater extent compared with the control mice, and the levels of RS3 + PLCSB were more close to the normal mice than PLCSB treated mice. Histopathological examination of sections of colon tissues showed that the RS3 + PLCSB group recovered well from colon carcinogenesis; however, the tissue sections of the stachyose + starch could reduce the necrosis degree. PLCSB significantly induced apoptosis in tissues of mice (*p* < 0.05) by up-regulating Bax, caspase-3, and caspase-9, and down-regulating Bcl-2. The expression of genes associated with inflammation-related *NF-κB*, *iNOS*, and *COX-2* genes, was significantly down-regulated, and *IκB-α* was up-regulated (*p* < 0.05). These results suggest that PLCSB is a potent preventive against *in vivo* colon carcinogenesis and that PLCSB with an RS3 carrier could increase the preventative effect in mice.

## 1. Introduction

As one of the main commercial fishes in the coastal waters of China, *Larimichthys crocea* swimming bladder contains rich protein, microelements and vitamins. Traditional medicine considers that it has good curative effect against diseases including amnesia, insomnia, dizziness, anepithymia and postpartum weakness [1]. Modern scientific research also suggests that *Larimichthys crocea* swimming bladder serves to remove free radicals and ward against cancers [2]. Swimming bladders are important balancing organs and the amount of polysaccharide in it constitutes as much as 10% of its weight. Polysaccharide is an important type of functional material. It has been proven that polysaccharide in swimming bladders can accelerate the healing of cuts and prevent infection as well as thrombus [3]. *In vitro* and *in vivo* experiments have proved that polysaccharide in lentinus edodes, lucid ganoderma and spirulina seaweed serves to prevent and cure colon cancer [4,5].

Resistant starch (RS) is the sum of starch and starch degradation products that are not absorbed in the small intestine since it is resistant to enzymatic digestion [6]. RS3 is a retrograded starch, formed on cooling in processed foods, including cooled cooked potato, bread and cornflakes [7]. RS appears to have a number of physiological effects, including weight control, prevention of diabetes, lipid reduction and promotion of inorganic salt absorption [8]. Functional polysaccharides would take effect in the infected part of colon cancer after being absorbed by the human body and some polysaccharides would be lost in the digestion process. Resistant starch would not be digested and absorbed in the stomach; it would be decomposed after entering the intestinal tract and utilized to wrap up functional material to decrease the loss of functional components in the digestion process, thus allowing more functional material to take effect after entering the intestinal tract. Therefore, the healthcare and cure effect of functional materials would be improved [9].

In the present study, the anticancer effect of polysaccharide of *Larimichthys crocea* swimming bladder (PLCSB) was tested. RS3 was used as a carrier for PLCSB and its preventative effect on colitis was further examined. The levels of inflammatory cytokines were used to determine the preventative effects on azoxymethane (AOM) and dextran sulfate sodium (DSS)-induced colon carcinogenesis in mice. Colon tissue histology was also used to determine the preventative effects *in vivo.* The mRNA and protein expression levels were determined for clarifying the preventative effects of PLCSB and RS3 + PLCSB.

## 2. Results and Discussion

### 2.1. Body Weight and Colon Observations

**Figure 1.**
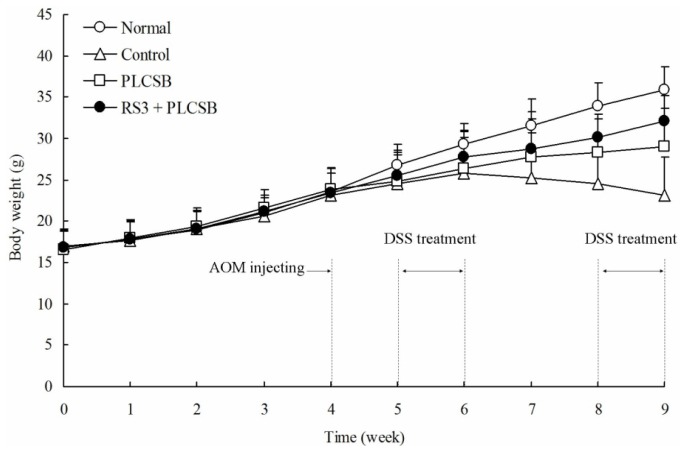
Changes in the body weight of AOM and DSS-induced colon cancer mice during the experiment.

Body weight is an important marker for colon cancer in mice; the body weight of AOM and DSS-induced colon cancer mice were lower than normal mice [10]. The normal mice had a normal diet and their body weight increased during the nine weeks of experiments. The body weights of the control mice with AOM and DSS-induced colon cancer were significantly decreased after six weeks. As shown in Figure 1, following the initiation of AOM and DSS-induced colon cancer, the body weights of the mice in the PLCSB and RS3 + PLCSB groups were significantly lower compared with those of the normal mice after four weeks, but higher than AOM and DSS-induced colon cancer control mice. RS3 + PLCSB treated mice could alleviate the weight loss compare to the PLCSB treated mice.

The total colonic length was significantly shorter in the AOM and DSS-treated mice (control group and sample treated group) compared with the normal mice as shown in Table 1 (*p* < 0.05). The normal mice had the longest colon length at 80.33 ± 3.86 mm and the colon length of control mice was the shortest (72.14 ± 5.21 mm). The total colonic length was longer in the RS3 + PLCSB-treated mice (77.31 ± 2.58 mm) than in the PLCSB-treated mice (75.47 ± 3.21 mm). AOM and DSS-induced colon carcinogenesis mice model is a staple research model for colon cancer. The increase in the ratio of colon weight to colon length was a consequence of apparent mucosal thickening and the formation of neoplasia [11]. The significant shortening of the colonic length in AOM and DSS-treated mice indicates that AOM and DSS contributed to the process of cancer changes in the colon. In particular, the colon weight and weight/length ratio were reduced slightly in the sample treatment groups compared to the control mice.

### 2.2. Effect of PLCSB and RS3 + PLCSB on Serum Levels of IL-6, IL-12, TNF-α and IFN-γ

**Figure 2.**
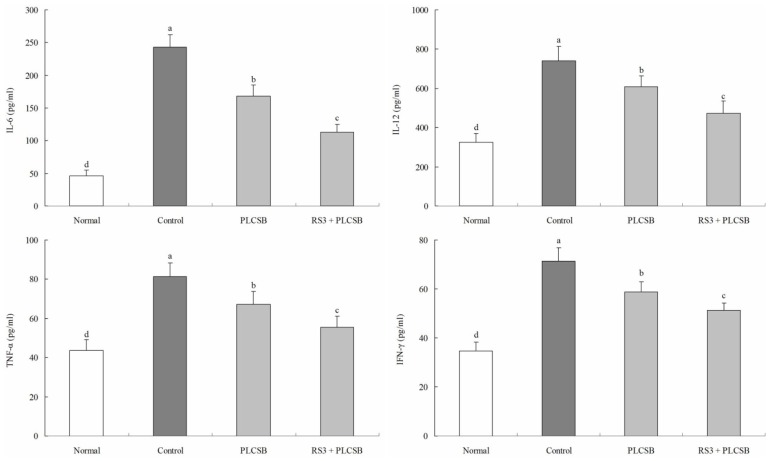
Serum IL-6, IL-12, TNF-α, and IFN-γ levels of mice with AOM and DSS-induced colon cancer treated with PLCSB and RS3 + PLCSB. PLCSB, polysaccharide of *Larimichthys crocea* swimming bladder; RS3, resistant starch 3; ^a–d^ Mean values with different letters over the bars are significantly different (*p* < 0.05) according to Duncan's multiple range test.

The IL-6 level of normal mice was 46.2 ± 8.9 pg/mL; however, in control mice the IL-6 level was significantly increased to 243.1 ± 18.6 pg/mL. The levels of IL-6 in mice fed with PLCSB and RS3 + PLCSB were 167.8 ± 17.5 and 112.6 ± 12.3 pg/mL, respectively (Figure 2). Control mice showed the highest IL-12 level in 741.3 ± 71.3 pg/mL, PLCSB and RS3 + PLCSB reduced the levels in 608.7 ± 55.2 and 474.3 ± 61.4 pg/mL, and the level of normal mice was lowest (324.7 ± 45.8 pg/mL). The TNF-α levels in the normal, control, PLCSB and RS3 + PLCSB treated mice were 43.7 ± 5.3, 81.2 ± 7.1, 67.2 ± 6.5 and 55.4 ± 5.7 pg/mL, respectively. The serum IFN-γ level in the mice in the RS3 + PLCSB treated group (51.4 ± 2.8 pg/mL) was significantly lower compared with those in the control (71.3 ± 5.5 pg/mL) and PLCSB treated group (58.7 ± 4.3 pg/mL), the level of normal mice was 34.7 ± 3.7 pg/mL. Inflammation is a critical component of tumor progression; cancers arise from infection and inflammation [12]. Cytokines are recognized to perform a major role in the immunopathogenesis of inflammatory bowel disease and the promotion of neoplastic transformation [11]. It is well known that the increased pro-inflammatory cytokines (IL-6, IL-12, TNF-α, and IFN-γ) amplify the inflammatory cascade and result in intestinal tissue damage in patients, as well as in animal models [13,14]. Cytokine receptors and the inflammatory cytokines IL-6, IL-12, IFN-γ and TNF-α play pathogenic roles in colon disease, lower levels of these cytokines were indicative of improved anticancer effects [15,16]. IL-6 is regarded as an important tumor-promoting factor in various types of human cancer; an increased expression of IL-6 is found in patients with colon cancer, where IL-6 levels are elevated in the serum of patients and in tumor tissue [17]. *IL-6* expression is regulated through the activation of several transcription factors such as NF-κB, C/EBPβ or AP-1. The regulation of *IL-6* expression through these transcription factors enables a rather unspecific up-regulation of this cytokine during nearly every type of inflammation-related cancer [18]. IL-12 through IFN-ã-dependent induction of the antiangiogenic factors interferon-inducible protein (IP) 10 and monokine induced by gamma interferon (MIG) contributes to tumor eradication [19]. Observations by Popivanova *et al*. [20] have revealed the crucial involvement of TNF-α in the initiation of chronic inflammation-mediated colon carcinogenesis; blocking of TNF-α reversed carcinoma progression, even after colon carcinoma was established. Drugs targeting TNF-α may be useful for the treatment of cancers, particularly those arising from chronic inflammation. In this study, we observed that the colonic levels of IL-6, IL-12, TNF-α, and IFN-γ in the AOM and DSS-induced colon cancer mice were markedly decreased by PLCSB and RS3 + PLCSB treatment. Based on this study, PLCSB showed a strong colon carcinogenesis preventive effect, and the PLCSB carried by RS3 could increase the anticancer effect.

### 2.3. Effects of PLCSB and RS3 + PLCSB on Histological Changes

**Figure 3.**
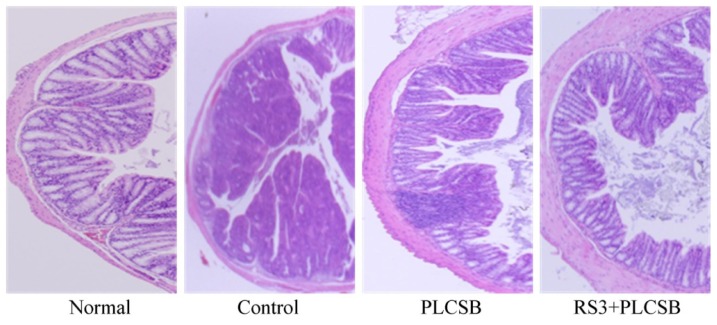
Microscopic observations of the effects of PLCSB and RS3 + PLCSB on histological damage in the colon tissues of mice with AOM and DSS-induced colon cancer (magnification, 40×). PLCSB, polysaccharide of *Larimichthys crocea* swimming bladder; RS3, resistant starch 3.

The H&E staining assay was used to evaluate the therapeutic effects of PLCSB and RS3 + PLCSB in AOM and DSS-induced colon cancer in mice (Figure 3). Normal mice did not show evidence of colonic inflammation, injury, or neoplasms. Nodular or polypoid colonic tumors were observed macroscopically in the colons of control group mice treated with AOM and DSS. The colonic tissues of mice receiving AOM and DSS evidenced mild to severe inflammation, characterized by crypt damage and inflammatory cell infiltration. The aforementioned mucosal thickening in the mice receiving AOM and DSS, as mentioned above, appeared to be attributable to the burden of colonic neoplasms. These phenomena did not distinctly differ between the PLCSB and RS3 + PLCSB fed groups. However, tissue sections from RS3 + PLCSB-treated AOM and DSS-induced colon cancer mice had more intact surface epithelium, normal colon cells and less inflammatory and neoplastic reactions than those in the PLCSB treated mice. Histopathological analysis is an important clinical standard used to diagnose colon cancer [21]. In addition, the histopathological examination of mouse colon sections is reported as an effective method to check the hepatoprotective activity against AOM and DSS-induced colon cancer in the mouse model [10]. From the sections examined in the present study, PLCSB and RS3 + PLCSB were observed to exert a preventive effect against AOM and DSS-induced colon cancer, and RS3 + PLCSB showed better anticancer effect than PLCSB treatment alone.

### 2.4. Effects of PLCSB and RS3 + PLCSB on Apoptosis-Related Gene Expression of *Bax*, *Bcl-2* and *Caspases*

**Figure 4.**
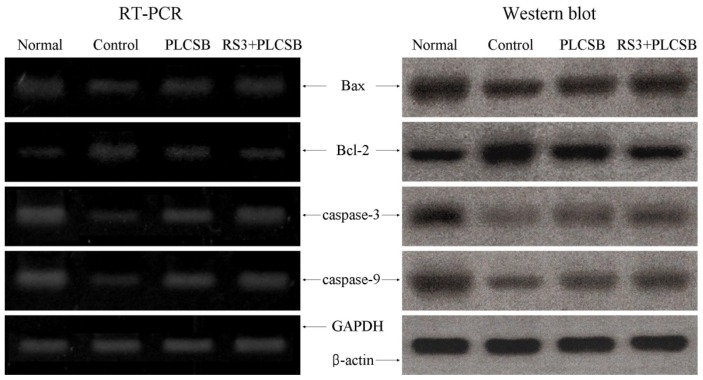
Effects of PLCSB and RS3 + PLCSB on mRNA and protein expression of *Bax*, *Bcl-2*, *caspase-3* and *caspase-9* in AOM and DSS-induced colon cancer mice. PLCSB, polysaccharide of *Larimichthys crocea* swimming bladder; RS3, resistant starch 3.

To elucidate the mechanisms underlying the colon carcinogenesis preventive effect by PLCSB and RS3 + PLCSB, expression of *Bax*, *Bcl-2*, and *caspase-3* and *caspase-9* in colon, tissues were measured by RT-PCR and Western blot analysis. As shown in Figure 4, expression of pro-apoptotic *Bax* and anti-apoptotic *Bcl-2* showed significant changes in the presence of PLCSB and RS3 + PLCSB. These results suggest that the PLCSB and RS3 + PLCSB induced apoptosis in the colon tissues via a *Bax* and *Bcl-2* dependent pathway. The mRNA expression levels of *caspase-3* and *caspase-9* were very low in untreated control mice, but significantly increased after the mice were fed with PLCSB and RS3 + PLCSB; mRNA and protein expressions of *caspase-3* and *caspase-9* was gradually elevated with RS3 + PLCSB. More specifically, apoptosis induction by RS3 + PLCSB was related to up-regulation of *Bax*, *caspase-9*, and *caspase-3*, and down-regulation of *Bcl-2* in terms of mRNA and protein expression. The effects of RS3 + PLCSB were greater compared with those of the PLCSB.

Apoptosis is a fundamental cellular event, and understanding its mechanisms of action will help harness this process for use in tumor diagnosis and therapy [22]. In a healthy cell, the anti-apoptotic protein *Bcl-2* is expressed on the outer mitochondrial membrane surface [23]. Because the *Bax* and *Bcl-2* genes are mainly expressed during apoptosis, we determined that these genes regulate apoptotic activity. Apoptosis results from activation of caspase family members that act as aspartate-specific proteases [24]. Caspases form a proteolytic network within the cell whereby upstream initiator caspases are activated early in the apoptotic process (*caspase-9*) and in turn activate other downstream caspases (*caspase-3*). Cytochrome c and procaspase-9 processing is highly dependent on *caspase-3*, placing this caspase in a central position as a regulator of essential apoptotic pathways in cancer cells [25]. *Caspase-3* was also reported to play a role as an amplifier of apoptotic signals (*i.e.*, by cleaving *Bcl-2*) [26].

### 2.5. Effects of PLCSB and RS3 + PLCSB on Inflammation-Related Gene Expression of *NF-κB*, *IκB-α*, *iNOS* and *COX-2*

**Figure 5.**
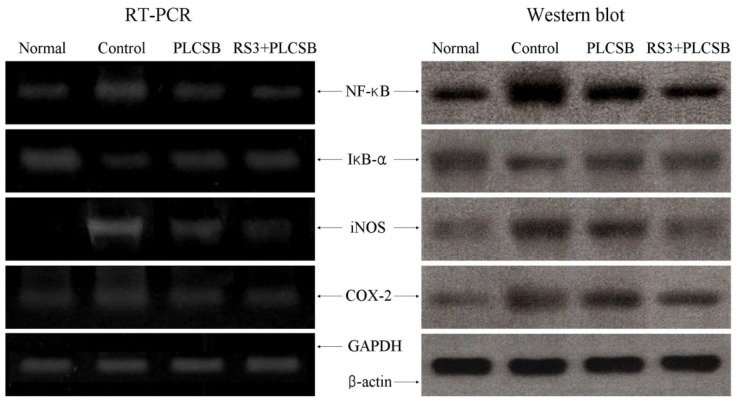
Effects of PLCSB and RS3 + PLCSB on mRNA and protein expression of *NF-κB*, *IκB-α*, *iNOS* and *COX-2* in AOM and DSS-induced colon cancer mice. PLCSB, polysaccharide of *Larimichthys crocea* swimming bladder; RS3, resistant starch 3.

The next experiments investigated whether the anticancer actions of PLCSB and RS3 + PLCSB were associated with inhibited expression of the inflammation-related genes *NF-κB*, *IκB-α*, *iNOS*, and *COX-2*. As shown in Figure 5, mRNA and protein expressions of *NF-κB* and *IκB-α* was reduced in colon tissues fed with PLCSB and RS3 + PLCSB. PLCSB and RS3 + PLCSB significantly modulated the expression of genes associated with inflammation. mRNA and protein expressions of *NF-κB* was decreased while *IκB-α* mRNA and protein levels were increased. Additionally, mRNA and protein expression of *COX-2* and *iNOS* were gradually decreased in the presence of the PLCSB dependent on use of RS3 as the carrier for PLCSB. Our findings indicate that PLCSB may help prevent cancer in the early stages by increasing anti-inflammatory activities. Overall, the results of this experiment showed that RS3 + PLCSB had a stronger anti-inflammatory effect on colon cancer than PLCSB.

Additionally, anticancer mechanisms underlying the effect of PLCSB and RS3 + PLCSB on AOM and DSS-induced colon cancer involve the induction of apoptosis by increasing the number of apoptotic bodies, regulating mRNA and protein expression of *Bax* and *Bcl-2*, and promoting anti-inflammatory effects by down-regulating *iNOS* and *COX-2* gene expression. *COX-2* has been suggested to play an important role in colon carcinogenesis, and NOS, along with *iNOS*, may be a good target for the chemoprevention of colon cancer [27]. Researchers have revealed the crucial involvement of the *IκB* kinase β/*NF-κB* (IKKβ/*NF-κB*) in colon carcinogenesis induced by combined treatment with AOM and DSS [20]. NF-κB is one of the most ubiquitous transcription factors, and regulates the expression of genes required for cellular proliferation, inflammatory responses, and cell adhesion [28]. NF-κB is present in the cytosol where it is bound to the inhibitory protein *IκB-α*. Following its induction by a variety of agents, NF-κB is released from *IκB-α* and translocates to the nucleus where it binds to the *IκB* binding sites in the promoter regions of target genes [29]. These mechanisms could be involved in the anticancer effects of PLCSB and RS3 + PLCSB in colon cancer, and RS3 + PLCSB could increase the anticancer effects in AOM and DSS-induced colon cancer.

## 3. Experimental Section

### 3.1. Polysaccharide *Larimichthys crocea* Swimming Bladder (PLCSB) Preparation

Wild Yellow Sea *Larimichthys crocea* were purchased from Shandong Province in China. Swimming bladder of *Larimichthys crocea* (1 kg) was dried by freeze-drying, and the dried samples were crushed. Three liters petroleum ether was added into swimming bladder of *Larimichthys crocea* and then reflux extraction was performed twice (1 h each time) at 60 °C to remove the protein; the residuals were gathered after filtration. Three liters absolute ethyl alcohol was then added and reflux extraction was performed for 3 h; the residuals without protein were filtrated and gathered. Finally, three liters water was added and the residuals were extracted at 60 °C for 2 h; filter liquid was collected. The crude polysaccharide of *Larimichthys crocea* swimming bladder was gained after evaporating [30].

### 3.2. Animals

Seven-week-old male C57BL/6 mice (*n* = 40) were purchased from the Experimental Animal Center of Chongqing Medical University (Chongqing, China). They were maintained in a temperature-controlled facility (temperature 23 ± 1 °C, relative humidity 50% ± 5%) with a 12 h light/dark cycle. The mice had unlimited access to a standard mouse chow diet and water.

### 3.3. Azoxymethane (AOM) and Dextran Sulfate Sodium (DSS)-Induced Colorectal Carcinogenesis Model

The mice were divided into four groups (*n* =10 each). The normal group mice received no treatment during the experimental period. The control group mice received no treatment during the first four weeks. The PLCSB group mice were fed with a mouse diet containing 1% PLCSB for four weeks; the RS3 + PLCSB group mice were fed with a mouse diet containing RS3 + PLCSB starch microspheres for four weeks, the mice diet also contained 1% PLCSB. Then the control, PLCSB and RS3 + PLCSB group mice were administered single intraperitoneal injections of 10 mg/kg bw azoxymethane (AOM, Sigma Co., St. Louis, MO, USA). After the injection for one week and four weeks, the mice received 2.5% dextran sulfate sodium (DSS, *M*_w_ 30,000–50,000, MP Bio., Solon, OH, USA) in their drinking water for one week. In the period of colon carcinogenesis induction, the PLCSB and RS3 + PLCSB group mice were continually fed with the sample diet. The body weight was recorded daily, and the colon length and weight were measured after dissection [10].

### 3.4. Analysis of Inflammation-Related Cytokines in Serum by Enzyme-Linked Immunosorbent Assay (ELISA)

For the serum cytokine assay, blood from the inferior vena cava was collected in a tube and centrifuged at 1100× *g*, 4 °C for 10 min. The serum was aspirated and assayed as described below. Concentrations of inflammatory-related cytokines IL-6, IL-12, TNF-α, and IFN-γ in serum were measured by ELISA according to the manufacturer’s instructions (Biolegend, San Diego, CA, USA). Briefly, biotinylated antibody reagent was added to 96-well plates, then supernatants of homogenized serum were added and the plates were incubated at 37 °C in CO_2_ for 2 h. After washing with PBS, streptavidin-horseradish peroxidase (HRP) solution was added and the plate was incubated for 30 min at room temperature. The absorbance was measured at 450 nm using a microplate reader (iMark; Bio-Rad, Hercules, CA, USA) [6].

### 3.5. Histological Analysis

The distal colons from each animal were subjected to histological examination. The colon tissues were fixed in 10% neutral-buffered formalin, dehydrated in ethanol and embedded in paraffin. Colon tissue sections (4 μm) were then cut and stained with hematoxylin and eosin (H&E) [31].

### 3.6. RT-PCR Assay

Total RNA from colon tissue cells was isolated using Trizol reagent (Invitrogen, Carlsbad, CA, USA) according to the manufacturer’s recommendations. The RNA was digested with RNase-free DNase (Roche, Basel, Switzerland) for 15 min at 37 °C and purified using an RNeasy kit (Qiagen, Hilden, Germany) according to the manufacturer’s protocol. cDNA was synthesized from 2 μg of total RNA by incubation at 37 °C for l h with avian myeloblastosis virus reverse transcriptase (GE Healthcare, Little Chalfont, UK) with random hexanucleotides according to the manufacturer’s instruction. Sequences of primers used to specifically amplify the genes of interest were as follows: 5′-AAGCTGAGCGAGTGT CTCCGGCG-3′ (forward) and 5′-CAGATGCCGGTTCAGGTACTCAGTC-3′ (reverse) for *Bax*; 5′-CTCGTCGCTACCGTCGTGACTTGG-3′ (forward) and 5′-CAGATGCCGGTTCAGGTACTCAG TC-3′ (reverse) for *Bcl-2*; 5′-CAAACTTTTTCAGAGGGGATCG-3′ (forward) and 5′-GCATACTG TTTCAGCATGGCA-3′ (reverse) for *caspase-3*; 5′-GGCCCTTCCTCGCTTCATCTC-3′ (forward) and 5′-GGTCCTTGGGCCTTCCTGGTAT-3′ (reverse) for *caspase-9*; 5′-CACTTATGGACAACTATGAG GTCTCTGG-3′ (forward) and 5′-CTGTCTTGTGGACAACGCAGTGGAATTTTAGG-3′ (reverse) for *NF-κB*; 5′-GCTGAAGAAGGAGCGGCTACT-3′ (forward) and 5′-TCGTACTCCTCGTCTTTCA TGGA-3′ (reverse) for *IκB-α*; 5′-AGAGAGATCGGGTTCACA-3′ (forward) and 5′-CACAGAACTGAGG GTACA-3′ (reverse) for *iNOS*; 5′-TTAAAATGAGATTGTCCGAA-3′ (forward) and 5′-AGATCACCT CTGCCTGAGTA-3′ (reverse) for *COX-2. GAPDH* was amplified as an internal control gene with the following primers: 5′-CGGAGTCAACGGATTTGGTC-3′ (forward) and 5′-AGCCTTCTCCATGGTC GTGA-3′ (reverse). Amplification was performed in a thermal cycler (Eppendorf, Hamburg, Germany). The polymerase chain reaction (PCR) products were separated in 1.0% agarose gels and visualized with ethidium bromide staining [32].

### 3.7. Western Blot Analysis

Total colon tissue protein was obtained with RIPA buffer as described [33]. Protein concentrations were determined with a Bio-Rad protein assay kit (Bio-Rad Laboratories Inc., Hercules, CA, USA). For the western blot analysis, aliquots of the lysate containing 30–50 μg protein were separated by sodium dodecyl sulfate-polyacrylamide gel electrophoresis (SDS-PAGE) and then electrotransferred onto a nitrocellulose membrane (Schleicher and Schuell, Keene, NH, USA). The membranes were subjected to immunoblot analysis and the proteins were visualized by an enhanced chemiluminescence (ECL) method (GE Healthcare). The cell lysates were separated by 12% SDS-PAGE, transferred onto a polyvinylidene fluoride membrane (GE Healthcare), blocked with 5% skimmed milk and hybridized with primary antibodies (diluted 1:1000). The antibodies against *Bax*, *Bcl-2*, *caspase-3*, *caspase-9*, *NF-κB*, *IκB-α*, *iNOS* and *COX-2* were obtained from Santa Cruz Biotechnology Inc. (Santa Cruz, CA, USA), then incubated with the horseradish peroxidase-conjugated secondary antibody (Santa Cruz Biotechnology Inc. (Santa Cruz, CA, USA) for 1 h at room temperature. The blots were washed three times with PBS-T and then developed by enhanced chemiluminescence (Amersham Life Science, Arlington Heights, IL, USA).

### 3.8. Statistical Analysis

Data are presented as the mean ± SD. Differences between the mean values for individual groups were assessed with a one-way ANOVA with Duncan’s multiple range test. Differences were considered significant when *p* < 0.05. SAS version 9.1 (SAS Institute Inc., Cary, NC, USA) was used for statistical analyses.

## 4. Conclusions

In summary, the colon carcinogenesis preventive effect of PLCSB and RS3 + PLCSB were evaluated by various *in vivo* experimental methods, including basic observations in mice (body weight, colon weight and colon length), serum cytokine assay, tissue RT-PCR, and Western blot assays. Mice consumed a mouse diet that included PLCSB or RS3 + PLCSB. PLCSB is partially decomposed by digestion prior to entering the colon; therefore, PLCSB is only partly absorbed in the colon, which reduces its efficacy. By contrast, PLCSB combined with RS3 is not absorbed in the stomach. After it enters into the colon, RS3 is broken down by intestinal bacteria, and PLCSB is released. As indicated by the experimental results, combining RS3 with PLCSB enables the full efficacy of PLCSB to be utilized. Compared with the direct use of PLCSB, this method produces better preventative effects against colon cancer.

## Figures and Tables

**Table 1.** Effects of PLCSB and RS3 + PLCSB treatment on colon lengths and colon weights of AOM and DSS-induced colon cancer mice.

Group	Colon length (mm)	Colon weight (g)	Colon weight/length (mg/cm)
Normal	80.33 ± 3.86 a	0.23 ± 0.03 d	28.63 ± 7.77 d
Control	72.14 ± 5.21 d	0.47 ± 0.08 a	65.15 ± 15.36 a
PLCSB	75.47 ± 3.21 c	0.35 ± 0.02 b	46.38 ± 6.23 b
RS3 + PLCSB	77.31 ± 2.58 b	0.30 ± 0.03 c	38.80 ± 11.63 c

PLCSB, polysaccharide of *Larimichthys crocea* swimming bladder; RS3, resistant starch 3;

a–d Mean values with different letters in the same column are significantly different (*p* < 0.05) according to Duncan’s multiple-range test.
